# Compromised Wound Healing in Ischemic Type 2 Diabetic Rats

**DOI:** 10.1371/journal.pone.0152068

**Published:** 2016-03-30

**Authors:** Peilang Yang, Qing Pei, Tianyi Yu, Qingxuan Chang, Di Wang, Min Gao, Xiong Zhang, Yan Liu

**Affiliations:** Department of Burn and Plastic Surgery, Shanghai Jiao Tong University School of Medicine, Ruijin Hospital, Shanghai 200025, China; University of Edinburgh, UNITED KINGDOM

## Abstract

Ischemia is one of the main epidemic factors and characteristics of diabetic chronic wounds, and exerts a profound effect on wound healing. To explore the mechanism of and the cure for diabetic impaired wound healing, we established a type 2 diabetic rat model. We used an 8weeks high fat diet (HFD) feeding regimen followed by multiple injections of streptozotocin (STZ) at a dose of 10mg/kg to induce Wister rat to develop type 2 diabetes. Metabolic characteristics were assessed at the 5th week after the STZ injections to confirm the establishment of diabetes mellitus on the rodent model. A bipedicle flap, with length to width ratio 1.5, was performed on the back of the rat to make the flap area ischemic. Closure of excisional wounds on this bipedicle flap and related physiological and pathological changes were studied using histological, immunohistochemical, real time PCR and protein immunoblot approaches. Our results demonstrated that a combination of HFD feeding and a low dose of STZ is capable of inducing the rats to develop type 2 diabetes with noticeable insulin resistance, persistent hyperglycemia, moderate degree of insulinemia, as well as high serum cholesterol and high triglyceride levels. The excision wounds on the ischemic double pedicle flap showed deteriorative healing features comparing with non-ischemic diabetic wounds, including: delayed healing, exorbitant wound inflammatory response, excessive and prolonged ROS production and excessive production of MMPs. Our study suggested that HFD feeding combined with STZ injection could induce type 2 diabetes in rat. Our ischemic diabetic wound model is suitable for the investigation of human diabetic related wound repair; especically for diabetic chronic wounds.

## Introduction

Type 2 diabetes mellitus is a complicated metabolic disorder characterized by abnormal glucose homeostasis due to anomalous insulin secretion and function [1]. Once a person gets diabetes, the risk that he may suffer from a diabetic foot ulcer could be as high as 25% [2]. Likewise, the probability of diabetes-related lower leg amputations during their lifetime exceeds 84% [3,4]. Foot ulcers causes both emotional and physical pain which can lead to productivity and financial losses [2,5].

Diabetic animal models that are spontaneously/genetically derived, chemically induced, or induced by nutrition/diet feeding combined with other procedures, have enormous contributions to the research of diabetes and its complications. We can understand the genetic and environmental factors that affect the development of diabetes and establish treatment strategies for human diabetes complications by mimicking the characteristic of diabetes and inducing diabetic complications in these models. However, no ideal type 2 diabetic animal model has been established until now. Each diabetic model simulates only part of features or complications of diabetes. For instance, the chemically induced model, mostly streptozotocin (STZ) induced, is a useful model for drug testing (eg PPAR agonist) [5] and studying the mechanisms of diabetic complications (e.g. diabetic nephropathy or retinopathy) [6,7]. Whereas the transgenic/knock-out model offers an ideal way to identify a single gene or protein in the development of diabetes, which, for example, makes it easier to dissect the complex genetics network of diabetes genes implicated in insulin resistance (e.g. IRS-1,IRS-2, GLUT-4) [8–10] or in lipid and glucose metabolism(e.g. PPARs) [11]. Unfortunately, no appropriate diabetic chronic wounds were available due to the complicated pathogenesis and wound situation. An ideal diabetic chronic wound model, in the macro-aspect, should include one or more following criteria: (1) Insulin resistance induced hyperglycemia; while insulin and relative insulin deficiency are optimal due to the fact that type 2 diabetic patients are the main populations with diabetic non healing wounds; (2) wound ischemic due to the vasculitis and dysfunction of microcirculation; (3) presence of neuropathy; (4) be combined with microbial infection or bacterial biofilm formation. Nowadays, the spontaneously/genetically derived diabetic model, like the obese/obese (ob/ob) and the obese diabetes/diabetes (db/db) mouse strains, were extensively used in the investigations of mechanisms and treatments related to diabetes-impaired wound healing. However, the usage and significance of these two diabetic models are greatly limited [12,13] because leptin itself plays an important role during wound healing [14]. The healing process of these gene modified mice can be interfered by the mutation of leptin and/or the leptin receptor. Some scientists claim that rodents maintained on a high fat diet (HFD) may be a better model for studying non-genetic risk factors induced obesity and metabolic disorder of human type 2 diabetes [15]. However, this model could barely provide any information on how high glucose levels affect wound repair because that simple dietary treatment could not induce significant hyperglycemic [16]. Additionally, their ‘sturdy’ beta cells could keep their endocrine function normal, or near normal, during their lives. STZ, on the other hand, is capable of causing obvious hyperglycemia due to the destruction of pancreatic beta cell function. Unfortunately, the deficiency of insulin resistance in STZ induced diabetic animal makes it an inefficient model for diabetic chronic wound research.

We tried to establish a diabetic animal model with both insulin resistance characteristics and obvious hyperglycemia by utilizing a combination of HFD feeding and STZ injection. We then made ischemic full thickness wounds on these diabetic rats and depicted their wound healing characteristics. Our results suggested that this ischemic type 2 diabetic wound model showed some diabetic ischemic wound characteristics and could be used in diabetes related wound research.

## Material and Methods

### Animals

60 male Wistar rats weighting 120~140g were purchased from Shanghai Laboratory Animal Center in the Chinese Academy of Sciences and housed at the Animal Science Center of Shanghai JiaoTong University, School of Medicine (SJTUSM). The animals were maintained under a 12h light/dark cycle at 22°C. The animal procedures were performed in accordance with the rules of the Animal Care Committee of SJTUSM, and all experimental protocols were approved by the SJTUSM Institutional Animal Care and Use Committee.

After 3 days of adjusting to the new environment, rats were randomly divided into two groups: the control group (Ctrl, n = 20) was fed a normal chow diet, whereas the diabetic group (DM, n = 40) was fed a HFD containing 60% (kcal) fat (soybean oil and lard that contains 0.95 mg cholesterol /g lard), 20% (kcal) carbohydrates and 20% (kcal) protein (Research diets, D12492, Research Diets Inc., New Brunswick, NJ) for 8wks. 8wks after HFD feeding, the rats of the diabetic group were fasted for 16h followed by multiple low dose intraperitoneal injections of STZ (10mg/kg BW dissolved in 0.1mM citrate buffer) for four consecutive days. Rats were then allowed to develop diabetes for 4 wks.

At 3, 6 or 8wks after feeding and 4wks after STZ injection, the body weight (BW) was recorded for all of the rats.

### Wounding procedures

HFD feeding and STZ injection induced diabetic rats (n = 40) were utilized in the following wounding experiments.

### The ischemic wounds

Rats (n = 20) were anaesthetized with a single intraperitoneal injection of thiopental sodium (40mg/kg BW). The backs of the rats were shaved, and the hairs were thoroughly removed by Nair. The outline of the flap was drawn using a surgical marker pen. The spine was set as the long axis and the flap extended from the scapulae to the iliac crests. 12x4cm double pedicle rectangular flap was then created by excising two sides which are parallel to the spine. The panniculus carnosus underneath the flap was then thoroughly removed. Four 0.9cm diameter full-thickness excision wounds were made on the flap at 5cm and 7cm from the top pedicle of the flap and 5 mm to the excision edge [17]. The flap was then repositioned and sutured with incisional edge normal skin. The wounds were covered with sterile gauze. The rats were then returned to their cages, maintained separately, and supplied with unrestricted food and water.

### The non-ischemic wounds

Rats (n = 20) were anaesthetized as mentioned above, four 0.9cm diameter full thickness excision wounds, expanding through the panniculus carnosus were made in the same place as described in ischemic group.

Immediately after the wound is created, a non-opioid analgesic, Tramadol, with dose of 20mg/kg, was administered subcutaneously to prevent post-operation pain. The signs of distress or discomfort, including increased aggression, reduced exploratory behavior, licking, biting, scratching and guarding were monitored for the first 48 hours after wounding. If obvious sign of distress or discomfort were observed, a bonus analgesic will be given. After completion of each experiment, the rat was euthanized using CO_2_.

### Measurement of wound closure

Rats were anaesthetized with thiopental sodium at day 1, 5, 9, 11 and 13 after the wounding. The wounds were photographed and drawn on transparent tracing paper. The paper was then scanned and the wound size was analyzed using ImageJ software. The unhealed rate was calculated by comparing the unhealed wound area to the original wound area. Initial (day 0) wound area was used as a baseline (Area_i_) and for each time point, healed wound area was calculated and expressed as Area_n_. Healing rates (%) = [Area_i_−Area_n_]/Area_i_ [18].

### Intraperitoneally glucose tolerance test (IPGTT)

Eight weeks after HFD or normal diet feeding, rats were fasted for 16hs and given an intraperitoneal injection of glucose with a dose of 2g/kg BW. Blood glucose (BG) levels were determined before and after 15, 30, 60, 90 and 120 mins of the glucose application.

### Blood collection and serum insulin, cholesterol and triglyceride measurement

Blood samples were collected at 8wks after HFD feeding and 4wks after STZ injection. A 1 ml blood sample was collected and the serum was extracted after a 15min centrifugation at 3000g. Fasting and non-fasting blood samples were collected over two consecutive days for a 4wk period. The serum insulin level was measured with a mouse/rat insulin ELISA kit, (Millipore, USA) and cholesterol and triglyceride levels were measured using spectrophotometric method on Beckman DX 800 (Beckman Coulter, Inc., Fullerton, CA) using standard laboratory protocol [19–21].

### Histological observation

The wounds, along with 5 mm of adjacent normal skin were harvested on days 5 and 11 after wounding. The wound tissue was excised in full depth including the subcutaneous fat tissue. The tissue was fixed in 4% paraformaldehyde and embedded in paraffin. Sections with 6~7um thickness were stained with hematoxylin and eosin (H&E) for histological and morphometric evaluation. Masson-trichrome staining [22] was used for collagen deposition evaluation. Morphometric measurements were made from sections through the center of the wounds, so as to obtain maximum wound diameter. The measurements were blindly performed three times, by studying the slides in different random sequence. The epithelial migration distance was measured using ImageJ software. Granulation tissue thickness was determined at the center of each section, vertically, and from the surface of granulation tissue to the junction boarder of dermis and subcutis. Values obtained in treated and control groups were compared and statistical analysis was carried out using the analysis of variance (IPP, USA).

### Hypoxia detection

80mins before the animals were scarified, pimonidazole hydrochloride adducts solutions were intraperitoneally injected at a dosage of 60mg/kg BW. The wound tissues were then harvest for pimonidazole detection [23].

### Immunohistochemistry

The wounds were harvest and fixed as mentioned above and sections were then deparaffinized, rehydrated, and washed in distilled water. The sections were placed in 95~98°C antigen retrieval citrate buffer in a container for 10~15 mins. Endogenous peroxidase activity was blocked by placing the sections in 3% hydrogen peroxide in methanol for 10 mins. Non-specific staining was blocked with normal goat serum, and the sections were incubated with monoclonal mouse anti-rat MMP-2/8/9 and TIMP-1 (Millipore), PECAM-1 (Santa Cruz) CD68 (abcam), MPO (R&D), 4 Hydroxynonenal (abcam) and Anti-pimonidazole MAb1 (Hypoxyprobe, Inc) overnight at 4°C. After washing, a HRP-labeled secondary antibody was applied for 1hr at room temperature then stained with diaminobenzidine, and counterstained with hematoxylin.

### Real time PCR

A total mRNA extraction was performed using Trizol. cDNA synthesis was performed using PrimeScript® RT reagent Kit Perfect Real Time using 1 mg RNA. The cDNA sample was subjected to PCR analyses using SYBR® Premix Ex Taq(Takara)™. The primers for the genes and the internal control (β-actin) are as follows: TNF-α: forward primer: 5′-GCTCCCTCTCATCAGTTCCA -3′, reverse primer: 5′-GCTTGGTGGTTTGCTACGAC-3′, IL-1β: forward primer: 5′-GTCAACTCCATCTGCCCTTC, reverse primer: 5′-TGTGGGTGGTATCCTCTGTG-3′, IL-2: forward primer: 5′-TCCCCATGATGCTCACGTTT-3′, reverse primer: 5′- TTTCCAGCGTCTTCCAAGTGA-3′, IL-10: forward primer: 5′-CAGACCCACATGCTCCGAGA-3′, Reverse primer: 5′-CAAGGCTTGGCAACCCAAGTA-3′ MMP2: forward primer: 5′-CCCGTTATGAGACCCTGAGC-3′, reverse primer: 5′-AGACCAATCGTGCCTCCATC-3′, MMP8: forward primer: 5′-TCCAGGTTACCCCACTAGCA-3′, reverse primer: 5′-AGTGACTCTGCGACTGACAAG-3′, MMP9: forward primer: 5′-AGGACGGTCGGTATTGGAAG-3′, reverse primer: 5′-GTACACCCACATTTTGCGCC-3′,TIMP-1forward primer: 5′-TCCTGGTTCCCTGGCATA-3′, reverse primer: 5′-ATCGCTCTGGTAGCCCTTCT-3′, TIMP-2: forward primer: 5′-GCATCACCCAGAAGAAGAGC-3′, reverse primer: 5′- TGACCCAGTCCATCCAGAG-3′, β-actin: forward primer: 5′-CTAAGGCCAACCGTGAAAAG-3′, reverse primer: 5′- CTAAGGCCAACCGTGAAAAG -3′. The samples were run on ABI 7500 Real-Time PCR System (Applied Biosystems) according to the following program: 95°C 30 secs; 95°C 5 secs x40; 62°C 34 secs. For data analysis, the DDC(T) method was applied as described previously.

### Western blot

The tissue was homogenized by pulverization in liquid nitrogen and transferred to tissue lysis buffer with a protease inhibitor cocktail following centrifugation at 12000R.P.M for 15 mins. The supernatant was removed and stored at -80°C. An equal amount of protein per lane (50μg) was separated by 5~12% SDS-PAGE and transferred to a polyvinylidene difluoride membrane. The membrane was blocked by 5% non-fat powdered milk in Tris-buffered saline with Tween-20 (TBST) and then incubated with anti-TNF-α (abcam), IL-6 and IL-10 (santa cruz) primary antibody in 5% non-fat milk in TBST overnight at 4°C. The membrane was then washed extensively with TBST, and then incubated with the secondary antibody for 1 hr at room temperature. Bands were visualized with enhanced chemiluminescence (Millipore). Relative quantities of protein were determined using a densitometer and presented in comparison with β-actin expression.

### Statistical analysis

Data is presented as mean ± SD. Data analysis was carried out using the student’s t test with the raw data. When multiple comparisons were performed, ANOVA was used.

## Results

### HFD induced obesity and insulin resistance

**Fig 1:**
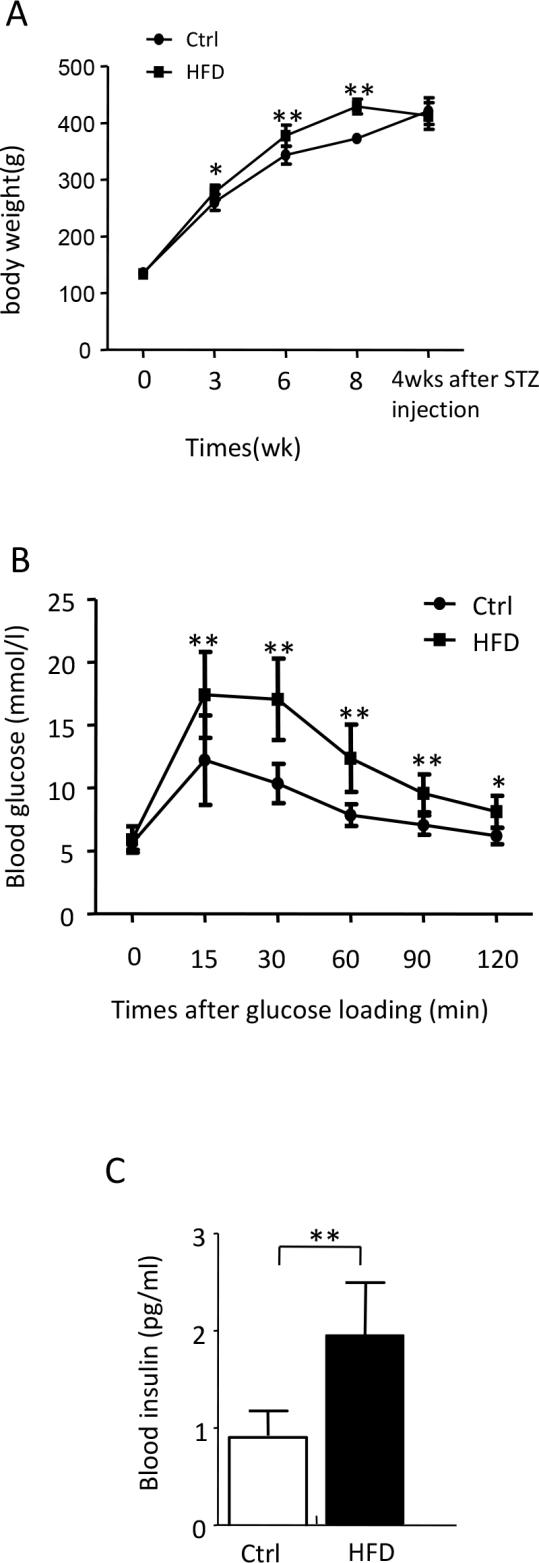
HFD induces obesity and insulin resistance. (A) Body weight was measured during 3wks, 6wks and 8wks of HFD/normal diet feeding and 4wks after diabetic induction in both control and diabetic group. (B) Both control and diabetic group animals were starved for 16 h. BG levels were determined using glucose meter at 0, 15, 30, 60, 90 and 120mins after given dose of glucose (2g/kg BW) were intraperitoneally injected. (C) Both control and diabetic group animals were starved for 16hs and 1ml of blood was collected individually. Serum insulin levels were determined using ELISA kit. Data are shown as the mean ± SD. *p <0.05, **p<0.*01*, *n = 60*.

HFD feeding animals began to show significant increase of BG after 3wks of feeding, comparing to the control group. The increase lasted until the beginning of diabetes induction which started at the eighth week of HDF feeding. During the first 4wks after STZ injection, rats showed a slight decrease in BW, while the rats of the control group continuously gained BW and had a higher BW than the HFD rats 4wks after diabetes induction (Fig 1A). Concomitant with the developing of HFD-induced obesity, a distinct insulin-resistance was obtained. IPGTT assay showed severely impaired clearance of the acute increase of blood glucose during the 2hrs blood glucose monitoring process (Fig 1B): the blood glucose levels in the HFD rats were significantly higher than that of the control rats at all time points. The staved serum insulin level of HFD rats, which was almost as twice as that of control rats, also confirmed these results (Fig 1C). (Data in S1 Table)

### Multiple STZ injections induce rats to developed stable type 2 diabetes with insulin resistance

**Fig 2:**
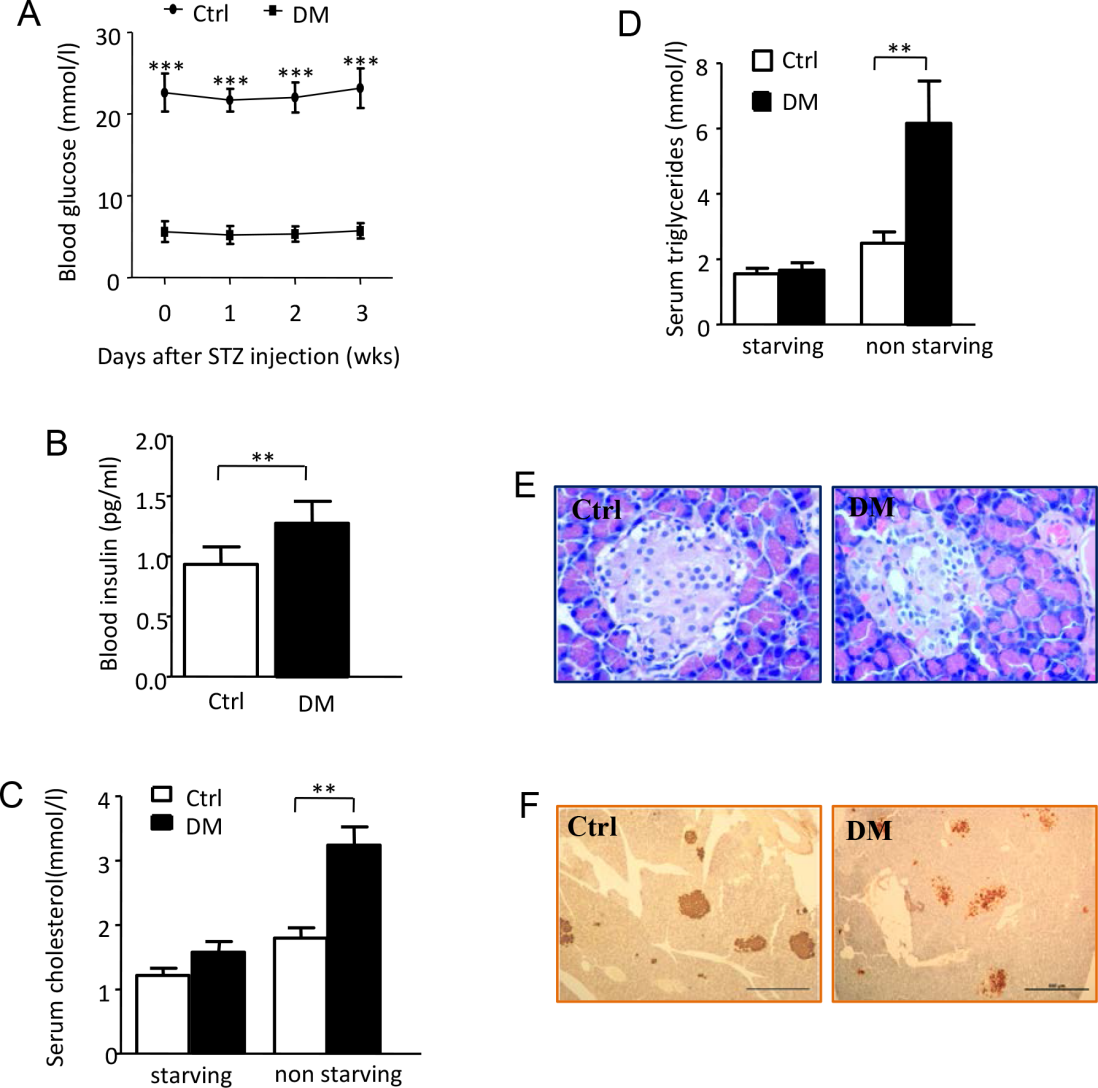
Multiple STZ injection induces rat developed stable type 2 diabetes with insulin resistant. (A) BG levels were determined every week after diabetic induction. Data are shown as the mean ± SD. ***p<0.*001*, *n = 60*. (B) 4wks after diabetic induction control group and diabetic group were starved for 16hs. serum insulin levels were determined by ELISA depicted as previously. (C-D) 4wks after diabetic induction serum starved and non-starved cholesterol and triglycerides levels of both groups were determined. (E, F) Pancreas tissues from both control and diabetic rats were collected when terminational experiment was performed and animal was euthanatized. E. Representative hematoxylin-eosin (H&E) staining section of pancreatic tissue. F. Representative images for immunohistochemical staining of insulin in the pancreatic tissue.

Following HFD feeding for 8wks, STZ induced a significant and continuous increasing in BG during the 4wks after injection and followed wounding healing process as well (Fig 2A). While the serum insulin was mildly reduced after 4wks of diabetic induction it was still higher than that of control group (Fig 2B). There is no significant difference of starving cholesterol and triglycerides level between the diabetic and control rats. However, a significant increase of non-starving cholesterol and triglycerides levels were found in the diabetic rats (Fig 2C and 2D). The histological examination of the pancreas showed smaller pancreatic islets in diabetic rats in comparison to the control rats. No obvious inflammatory cell infiltration was found in either group (Fig 2E). The presence of insulin inside the pancreas in the diabetic rats suggests that although the pancreas islets were partially destroyed by STZ, the remaining pancreas islets still performed their endocrine functions (Fig 2F). (Data in S2 Table)

### Performing of bipedicled flap induced ischemia and impaired wound healing

**Fig 3:**
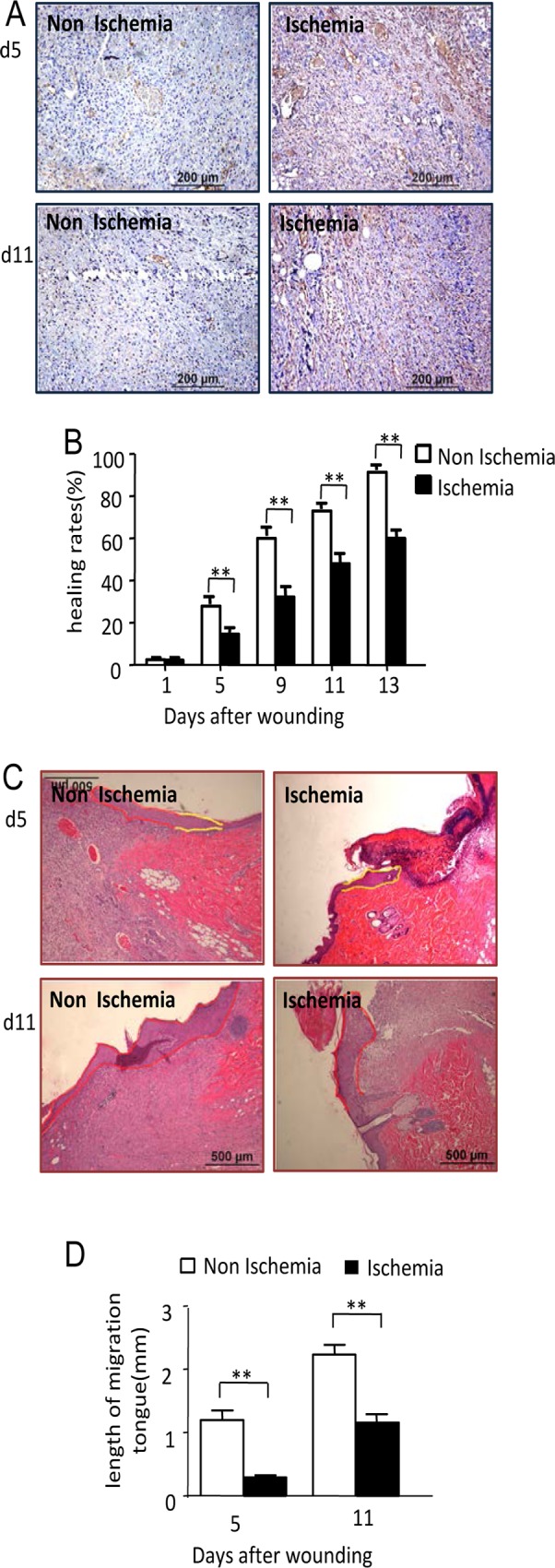
Performing of bipedicled flap induced ischemia and impaired re-epithalialization and wound healing. (A) 80mins before animals were scarified, pimonidazole hydrochloride adducts solutions were intraperitoneal injected at a dosage of 60mg/kg BW. The wound tissues were then harvested. Pimonidazole hydrochloride adduct were determined with an anti-pimonidazole antibody using immunohischemistry method. Scale bars = 200μm. (B) Wounds size were record at day 1, 5, 9, 13 after wounding using transparent tracing paper. Healing rate of wounds was calculated. Data are shown as the mean ± SD. **p<0.*01*. (C) Representative H&E stained sections showing shorter migrating tongue in ischemic wounds at day 5 and 11 after wounding. Scale bars indicate 500 μm. (D) Data was obtained from 8 wounds (n = 4), and shown as the mean value ± SD. Statistics are shown as comparisons between the non-ischemic wounds and ischemic wounds. **p<0.*01*. The proliferation zone was highlighted in yellow line and the migration tongues were depicted in red line.

To make wounds ischemic, a bipedicled flap with the length to width ratio of 1.5 (12cm/4cm/2) was made on the back of diabetic rats. A pale appearance accompanied by a low skin temperature demonstrated the presence of ischemia. However, no signs of necrosis, such as incisal margin blackness, dryness, eschar, etc. were found during the whole healing process. Wound ischemia was further confirmed using a hypoxia marker, also called hypoxyprobe, a pimonidazole hydrochloride adduct. Pimonidazole hydrochloride adduct levels in the ischemic wound were significantly higher than the non-ischemic wound at day 5 and day 11 after wounding (Fig 3A).

We then investigated the wound healing characteristics under this ischemic condition and compared them with non-ischemic diabetic wounds. Healing rates of the wounds showed an obvious impairment of wound healing: at day 5, 9, 11 and 13 after wounding; healing rates in the ischemic wounds were significantly lower than the non-ischemic wounds (Fig 3B). The healing days of the ischemic wounds were 22.17±0.37 days which is significant longer than the 16±0.31 days of the non-ischemic wounds. Keratinocyte migration was estimated using the length of migration tongue: at day 5 and 11 after wounding, the length of migration tongue in non-ischemic wounds was 1215.65 ±152.01μm and 2250.21±153.36μm, while in the ischemic diabetic wounds, the length of migration tongue was 307.91±30.83μm and 1174.70±133.86μm (Fig 3C and 3D). This suggests severely impaired keratinocyte migration in ischemic diabetic wounds. ((Data in S3 Table)

**Fig 4:**
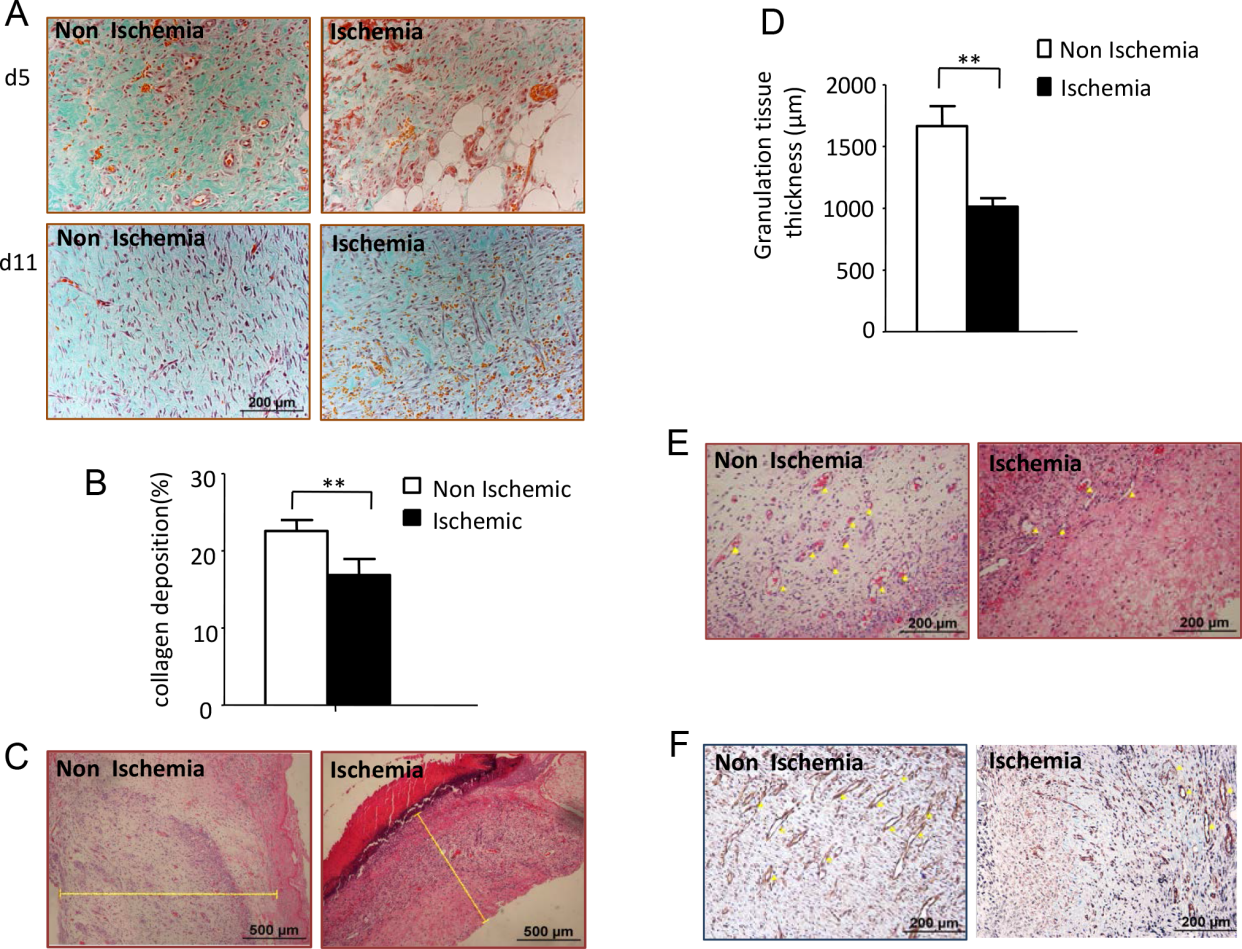
Performing of bipedicled flap induced impaired granulation tissue formation and wound healing. (A) Representative H&E stained sections showing thinner granulation tissue thickness in ischemic wounds at day 5 after wounding. Scale bars indicate 500μm. (B) Data was obtained from 12 wounds (n = 6), and shown as the mean value ± SD. Statistics are shown as comparisons between the non-ischemic wounds and ischemic wounds. **p<0.*01*. (C) Masson-trichrome staining showing collagen deposition of non-ischemic and ischemic wound at day 5 and 11 after wounding. (D) Collagen deposition rate were calculate using IPP software. Data was obtained from 12 wounds (n = 6), and shown as the mean value ± SD. **p<0.*01*. (E) Representative H&E stained sections and immunohischemistry stained with anti-PECAM-1 antibody showing angiogenesis of both non-ischemic and ischemic wounds at day 5 after wounding. Scale bars = 200μm.

Sparse collagen deposition was found in the ischemic wounds, which was most obvious at day 5 after wounding. Large numbers of fibroblast-like cells, surrounded by thin, discrete and scattered collagen fibers, were found on the ischemic wounds at day 11 after wounding. Notably different, fibroblast-like cells along with surrounding dense and abundant collagen were found on the non-ischemic wounds at the same time frame (Fig 4A). The collagen deposition rate of the ischemic wounds at day 5 was 16.86±2.09%, whereas the rate of the non-ischemic wounds was 22.56±1.42% (Fig 4B). The granulation tissue thickness in the ischemic wounds at day 5 after wounding was significantly lower than the non-ischemic group (Fig 4C and 4D). Using H&E (Fig 4E) and immunohistochemistry staining, utilizing a specific endothelia cell marker PECAM-1 (Fig 4F), we observed remarkably delayed angiogenesis in the ischemic wounds at day 5 after wounding compared with the non-ischemic wounds. (Data in S4 Table)

### Ischemia induced exorbitant wound inflammatory response

**Fig 5:**
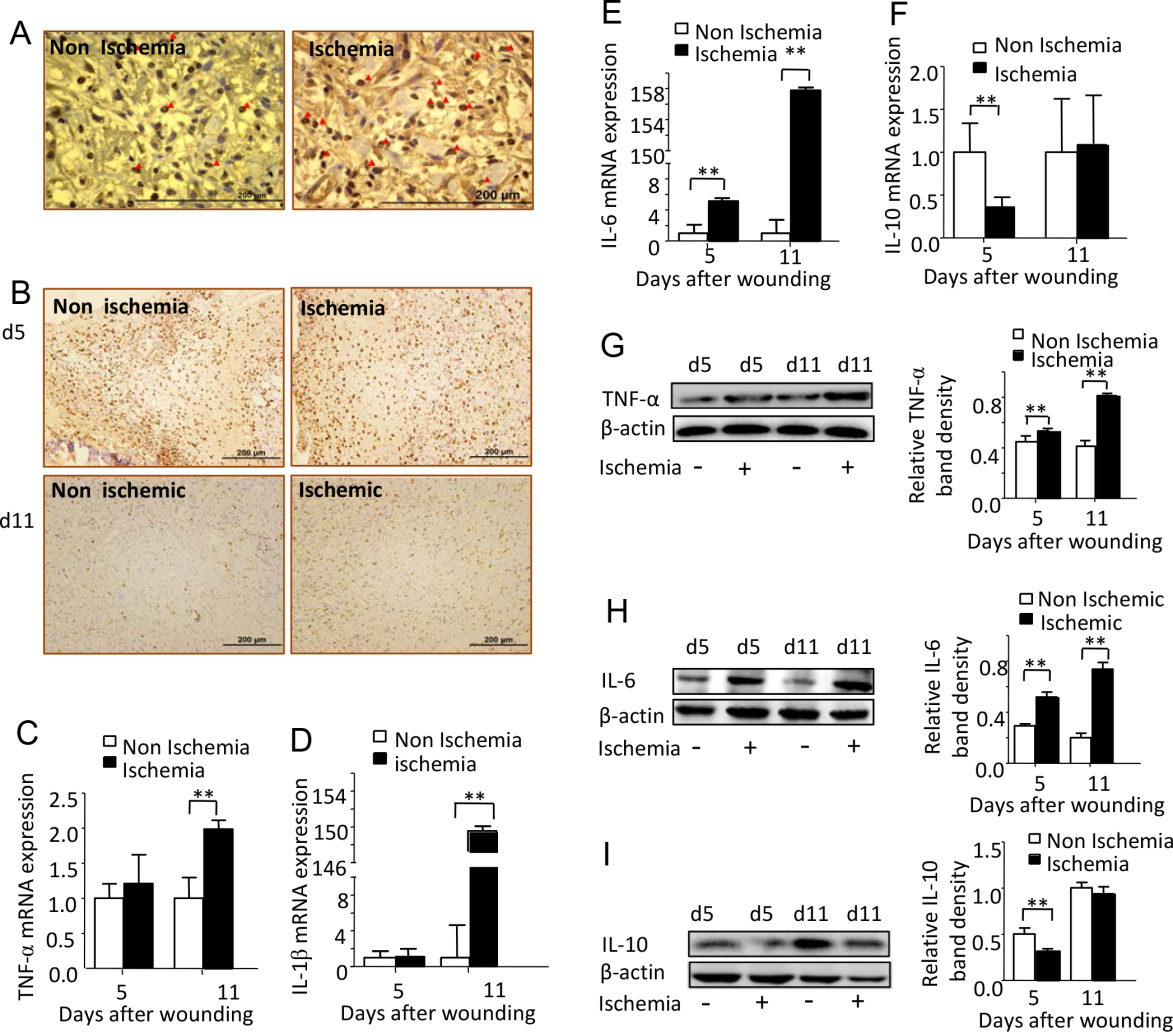
Ischemia induced exorbitant wound inflammatory response. (A) Representative images of MPO immunochemistry staining for both non-ischemic and ischemic wounds at day 5 after wounding. Scale bars = 200μm. (B) Representative images of immunohischemical staining for CD68, a specific marker of macrophage. Scale bars = 200μm. (C-F) Real time PCR analyze TNF-α, IL-6, IL-1β and IL-10 RNA expression (fold change) of non-ischemic and ischemic wounds at day 5 and 11 after wounding. Data was obtained from 12 wounds (n = 6), and are shown as the mean ± SD. **p<0.*01*. (G-I) Western blot analyze of wounds TNF-α, IL-6 and IL-10 at day 5 and 11 after wounding. Data was obtained from 12 wounds (n = 6), and are shown as the mean ± SD. *P<0.*05*, **p<0.*01*.

To explore whether our ischemic diabetic wound showed an excessive inflammatory response, normally found in diabetic ischemic chronic wounds, we first detected wound neutrophils and macrophages infiltration by labeling MPO and CD68, specific enzyme or marker for these two inflammatory cells. Larger amount MPO expression cells were found on day 5 ischemic wounds, suggesting more neutrophils infiltration induced by ischemia (Fig 5A). More macrophages were also found in the ischemic diabetic wounds. Furthermore, macrophage resolution was also impaired, shown by the existing of much more CD68 positive cells on wounds at day 11 after wounding (Fig 5B).

We then detected the inflammatory mediators; the main courier of wound inflammatory response. The RNA expression of TNF-α (Fig 5C) and IL-1β (Fig 5D) in the ischemic wounds was significantly higher than in the non-ischemic wounds at day 11 after wounding, while IL-6 (Fig 5E) and IL-2 (data not shown) in the ischemic wounds were significantly higher than that in the non-ischemic wounds at both day 5 and day 11 after wounding. Significantly low expression of anti-inflammation cytokines IL-10 was found in the ischemic wounds when compared with the non-ischemic wound at day 5 (Fig 5F). IL-4 was undetectable in the ischemic wounds at day 5 after wounding and showed a very low level of expression at day 11 after wounding (data not shown). Western blot showed a similar expression pattern as RNA expression for TNF-α, IL-6, and IL-10 (Fig 5G–5I). (Data in S5 Table)

### Ischemia induced excessive and prolonged ROS production and excessive production of MMPs

**Fig 6:**
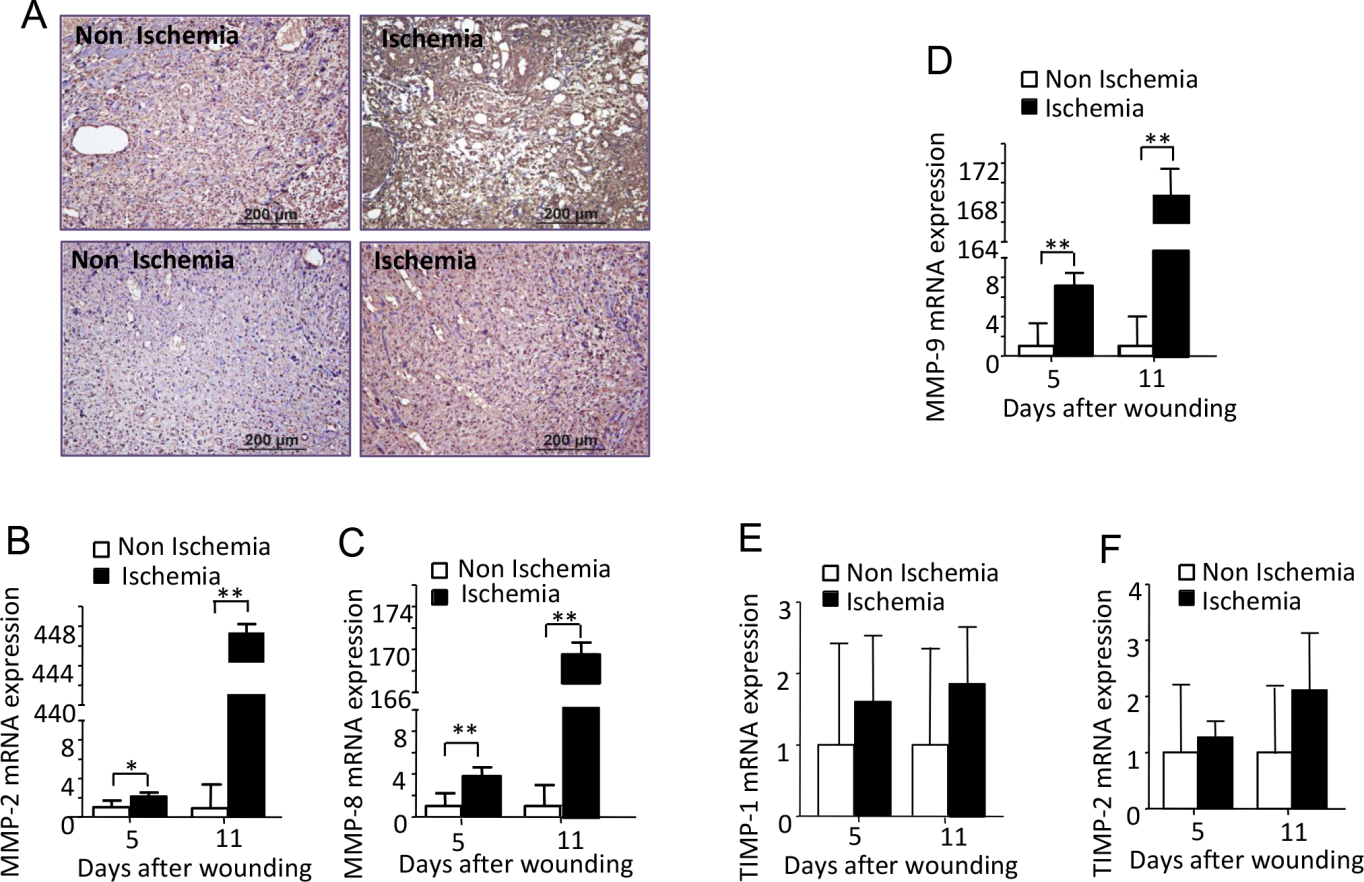
Ischemia induced excessive and prolonged ROS production and excessive production of MMPs. (A) Representative images for immunohischemical staining of 4-HNE, a specific marker of ROS Non-ischemic and ischemic wounds at day 5 and 11 after wounding were collected in 5day and 11day wounds of. Scale bars = 200μm. (B-F) Real time PCR analysis (fold change) of premiers for MMP-2, MMP-8, MMP-9, TIMP-1 and TIMP-2 in 5day and 11day wounds of non-ischemic and ischemic wound. Bars indicated the mean± SD obtained from 12 wounds (n = 6). *P<0.05, **p<0.01.

**Fig 7:**
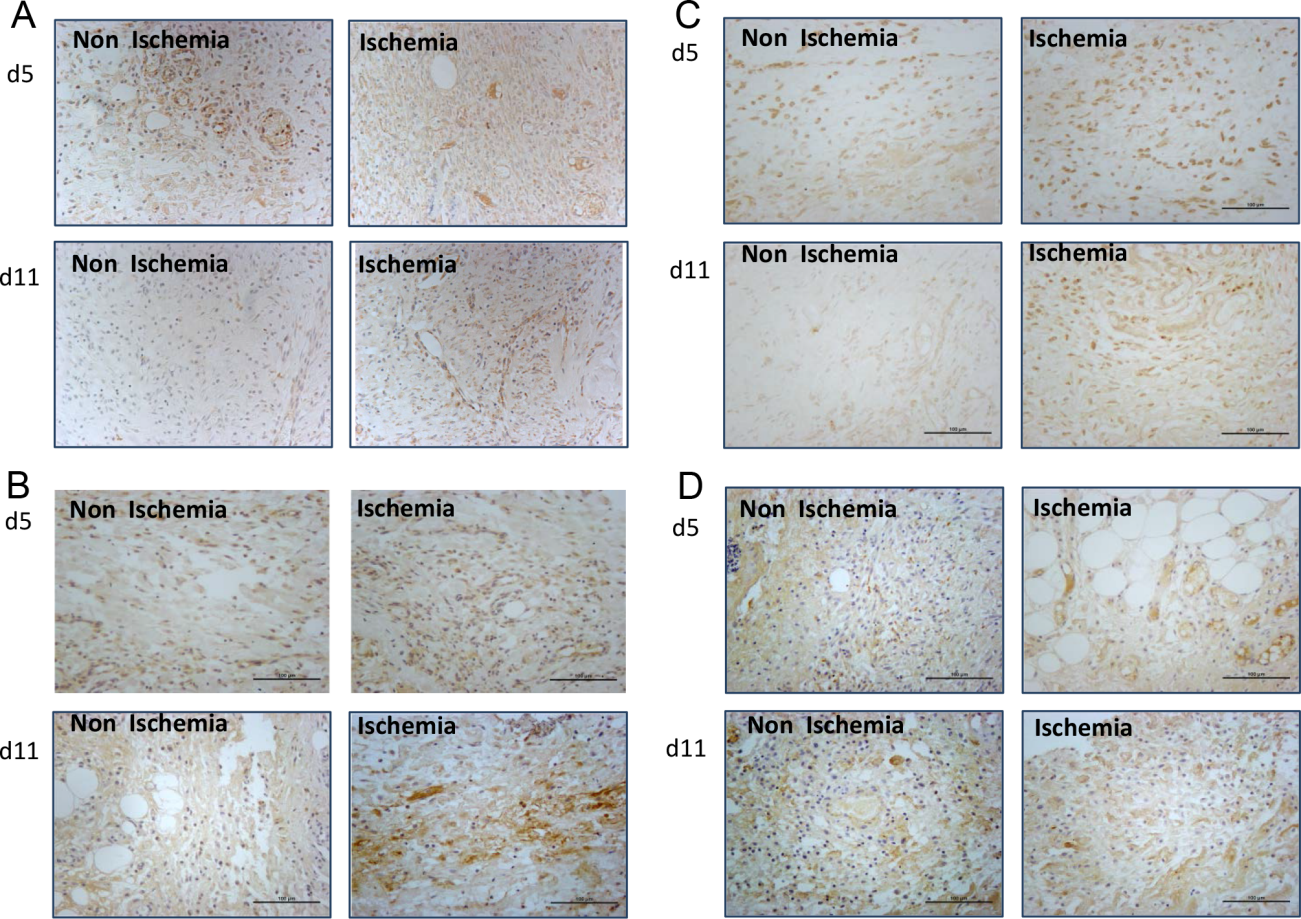
Ischemia induced excessive and prolonged ROS production and excessive production of MMPs. (A-D) Representative images for MMP-2, MMP-8, MMP-9 and TIMP-1 immunohischemical staining of non-ischemic and ischemic wounds at day 5 and day11 after wounding. Scale bars = 200μm.

The expression of 4-hydroxynonenal (4-HNE), a byproduct of lipid peroxidation during oxidative stress, was found to be much higher in the ischemic diabetic wounds at day 5 and day 11 after wounding compared with the non-ischemic diabetic wounds (Fig 6A). Using real-time PCR, we detected an extraordinary increase in the RNA expression of MMP-2, 8 and 9 in the ischemic wounds at day 5 and day 11 after wounding. Whereas, the TIMP1 and TIMP-2 RNA expression level did not show a significant difference between the two types of wounds (Fig 6B–6E). (Data in S6 Table). The protein levels of MMP2, MMP8, MMP9 and TIMP1 detected by immunochemistry showed a good correlation with their RNA expression (Fig 7A–7D).

## Discussion

Diabetic ulcers are the major cause for hospital admissions and amputation for diabetes patients [24]. Finding a diabetic chronic wound model that precisely mimics the pathological characteristics of diabetic wounds is in persistent demand. Here, we introduced a type 2 diabetes rat model and studied ischemic excision wounds healing process using this model. We found that: (1) a combination of HFD feeding for 8wks and 4 consecutive days of low dose STZ injections induced rat to develop type 2 diabetes with noticeable insulin resistance, persistent hyperglycemia, moderate degree of insulinemia, as well as high serum cholesterol and triglyceride levels. (2) The excision wounds on the double pedicle flap showed diabetic chronic wounds features: including relative hypoxia wound environment, excessive inflammatory response, impaired re-epithelialization and angiogenesis, reduced extracellular matrix (ECM) deposition, and delayed healing time. These results suggested that our diabetic wounds model exhibits many diabetic chronic wound characteristics and could be considered a favorable animal model for diabetic wound healing research and for developing new therapies for diabetes related chronic wounds.

STZ is a toxic nitrosurea derivative isolated from streptomyces achromogenes that specifically acts on insulin-producing β-cells in the pancreatic islets. The type of diabetes induced by STZ injection has been controversial for decades. It is generally accepted that STZ induces type I diabetes [25]: A single large dose of STZ induce type I diabetes by its direct toxic effects including formation of nitric oxide, alkylation of mitochondrial DNA and proteins [26]. Multiple low doses of STZ, combined T cell mediated autoimmune effect with the direct toxic effects of STZ, synthetically induced type I diabetes [27]. STZ alone [28] or combined with nicotinamide [29] induced type 2 diabetes also has been reported. Some researchers tend to set STZ-induced diabetic model as an uncategorized model since it does not bear obvious autoimmune features [30]. Variations of STZ injection protocols were used in individual research institutions in terms of dosage, route of injection, and insulin compensation manner. For diabetic rats induction, the dosage of STZ from 30mg/kg to 80mg/kg [31]. In the pilot experiment for diabetes induction, we tried single injection STZ with a dose of 30mg/kg. We observed a large proportion of animal death with obvious hemoglobinuria, which suggested the possibility of kidney injury. Kidney injury is the main acute side effect of STZ and the main cause of animal death during the diabetes induction process [32]. To avoid this severe side effect, we conducted multiple STZ injections with doses as low as 10mg/kg. The obvious and continuous increase in BG suggested that STZ, even with very low dose, is capable of inducing pancreatic β-cell destruction in rats. Smaller sizes of pancreatic islets, fewer inflammatory cell infiltrations, and secreted insulin particles found inside pancreas explicitly suggested that 10mg/kg of STZ caused partial damage of pancreas islets. The remaining endocrine functions of the pancreas along with HFD induced insulin resistance, provided this HFD diet and chemical induced animal model with insulin resistance, high BG levels and moderate hyper-insulinemia; which is in accordance with the definition of type2 diabetes [33].

Ischemia has enormous effects on wound healing. Some researchers suggested that all diabetic foot ulcers should be considered as ischemic unless there is definite evidence to the contrary. The temporary hypoxia and ischemia that happened in the normal wound healing process could trigger repairing and show constructive effects on wound healing [34]. Whereas prolonged ischemia may possibly inhibit healing, since the oxygen and other nutrients are carried in and metabolic waste is carried out by blood flow. Sustained ischemia causes the build-up of metabolic wastes; accumulated waste, combined with low oxygen level and less nutrition supply may induce mitochondrial, cell, even tissue damage [35–38]. Ischemia could also induce mitochondrial oxidative phosphorylation impairment with subsequently reduced ATP production. As a consequence of low ATP production, the key energy dependent ion exchangers such as Na^+^/k^+^-ATPase or Ca^++^ ATPase will not function, which would eventually lead to intracellular calcium ions overload and further induces membrane disruption and ROS production as well. Calcium ions overload, ROS production, cell damage and necrosis could trigger an inflammatory cascade via various signaling pathways [37,39]. In other words, ischemia is the fuse for wound tissue damage and exorbitant wound inflammatory response. It is also one of the chief culprits of impaired healing. Skin flaps are one of the classic approaches to induce ischemia. There are various types of flap models used for ischemic wound research: U shape peninsular incision with 2:1 length-to-width ratio, which can cause nearly 40% massive necrosis of the flap [40]; double pedicle ischemic model on white pigs and rats, which have a length-to-width ratio of 1.5:1, exhibited a severe ischemic state [41]. Utilizing our type 2 diabetic animal model and taking examples from the aforementioned references, we created a double pedicle flap to induce a relative ischemic condition for the wound healing study. During the early days after double pedicle flap performance, we found a pale appearance and lower skin temperature in the flap site which suggested the occurrence of ischemia. The ischemic state was further confirmed by the expression of pimonidazole hydrochloride adducts in wound area. Significantly higher pimonidazole hydrochloride adduct levels at day 5 and day 11 after wounding, suggested that ischemic condition could last for at least 11days.

Abnormal inflammatory response is a noticeable characteristic and the main cause of impaired diabetic wound healing. Compared with non ischemic wounds, the resolution of neutrophils and macrophages on our ischemic wounds were significantly delayed: massive macrophages still existed in wounds area even 11 days after wounding; whereas scarce macrophages were found in the non-ischemic wounds at the same time point. Significantly higher pro-inflammatory cytokines expression and relatively low anti-inflammatory cytokines expression in our ischemic wound suggested that ischemia induced a pro-inflammatory response. Dysfunction in inflammatory cells, for example, the dysfunction of phagocytosis, could cause necrotic tissue and bacterial colonization in the wounds not be removed in time [42–44], and then lead to delayed and lasting inflammatory cell resolution. The sustained existence of inflammatory cells secretes a massive amount of inflammatory cytokines [45] and consecutively recruit more inflammatory cells to the wound sites. Overloading inflammatory cells generates a pro-inflammatory environment which triggers more tissue damage, the degradation of ECM, and denaturation of growth factors by secreting ROS, pro-inflammatory cytokines and MMPs [46,47]. Another cause of the dysfunction of ECM deposition could be the over production of advanced glycation end products (AGEs) in diabetic individuals which induce fibroblast apoptosis and dysfunction of ECM production [48]. The imbalance of deposition and degradation of ECM affects keratinocyte and endothelial cell function, eventually leads to impaired re-epithelialization and angiogenesis [49,50]. Besides impaired re-epithelialization, hyper-proliferation of epithelium can also be found in ischemic wounds [51], which was also observed in our animal model during the healing process. All these factors combined cause the wound to fall into an inflammatory trap, preventing it from moving forward to the next step of the normal healing process, and ultimately could develop into a diabetic impaired or non-healing wound.

Several ischemic models have been reported. Both ischemic rabbit ear model [52] and rat hindlimb ischemia model [53] utilize arteries ligation technique. However, their feasibility is limited due to the limitation of wounds number. Transverse rectus abdominal myocutaneous (TRAM) flap model, tube flap model [54], and general flap model [17] can induces skin wound ischemia. By disrupting epigastric vessels, TRAM flap induces severe ischemia shown by necrotic skin wounds. The technical difficulty and the fact that only one wound can be made each time limit the model’s practicability. The wounds number is also limited in tube flap model. Skin flap model induces ischemia with or without necrosis, depending on the severity of perfusion occlusion. In brief, the length and width ratio of flap, and whether using the inclusion of silicone sheets to inhibit vascular in-growth from underlying vessels or not are two determinants of severity of ischemia. General flap is widely used because multiple wounds can be made. Combination of general flap and diabetic animal model makes it possible to study diabetic ischemic wound healing. Gupta et al [55] has reported a diabetic ischemic rat wound model using Zucker diabetic fatty rats that develop obesity and diabetes on a high fat diet due to a mutation in the Leptin receptor. This model showed typical type II diabetes characteristics demonstrated by hyperglycemia and insulin resistance. Delayed healing with impaired angiogenesis and poorly developed lymphatics and peripheral nerve fibers was also observed. However, because of the role of leptin on accelerating wound healing, the effect of leptin or leptin receptor deficiency on healing process cannot be neglected. Compared with leptin or leptin receptor mutated diabetic animal model, our model showed the superiority by endowing animal typical insulin resistance without the interference of leptin deficiency on healing.

Although our model mimics many features of diabetic wounds, we are aware of the limitations of our work. First of all, the ischemia is caused by transient deprival of blood perfusion due to the disruption of the blood supply, not chronic and persistent insufficient of blood supply caused by artery atherosclerosis and microangiopathy found in diabetic patients. The ischemia will gradually relieve and eventually recover completely if the blood vessels in the flap connect with the vessel network in adjacent normal skin. Secondly, we did not detect the incidence of peripheral neuropathy in this animal model. Peripheral neuropathy is another important factor in diabetic ulcer development. Some previous researches revealed that mice that received 16 wks of 60% HFD could develop a nerve conduction deficit, thermo and mechanical hypoalgesia and tactile allodynia [56,57]. Another report showed that 3wks of STZ injection induced slower motor nerve conduction velocity in rats [58]. Before operative procedures, our animals would have already received 13wks of HFD feeding, and exhibited a maintained high BG state for 5wks. It is highly possible that peripheral neuropathy may also exist. It will be beneficial to further confirm the presence of peripheral neuropathy in this diabetic animal. Considering the vital role of microbes in developing diabetic ulcers, introducing certain species of bacteria to the wounds will be another option for improving the significance of this model. Finally, knowing the chronicity of diabetes, there will be no doubt that many chronic pathological factors, including the accumulation of AGEs, diabetic neuropathy, and diabetic angiopathy will exert enormous effect on wound healing. To elucidate the long term effects of these pathological factors on healing, long duration of diabetic state of animal model will be indispensable, and that will be part of our future work.

## Supporting Information

S1 Table Supporting Information for Fig 1. (XLSX)

S2 Table Supporting Information for Fig 2. (XLSX)

S3 Table Supporting Information for Fig 3. (XLSX)

S4 Table Supporting Information for Fig 4. (XLSX)

S5 Table Supporting Information for Fig 5. (XLSX)

S6 Table Supporting Information for Fig 6. (XLSX)
